# Dissecting the Interaction of FGF8 with Receptor FGFRL1

**DOI:** 10.3390/biom10101399

**Published:** 2020-10-01

**Authors:** Lei Zhuang, Monique Vogel, Peter M. Villiger, Beat Trueb

**Affiliations:** 1 Department for BioMedical Research, University of Bern, 3008 Bern, Switzerland; lei.zhuang@dbmr.unibe.ch (L.Z.); monique.vogel@dbmr.unibe.ch (M.V.); 2 Department of Rheumatology, University Hospital, 3010 Bern, Switzerland; peter.villiger@insel.ch

**Keywords:** fibroblast growth factor (FGF), fibroblast growth factor receptor (FGFR), FGFRL1, FGF8, kidney development, mesenchymal-to-epithelial transition (MET)

## Abstract

In mammals, the novel protein fibroblast growth factor receptor-like 1 (FGFRL1) is involved in the development of metanephric kidneys. It appears that this receptor controls a crucial transition of the induced metanephric mesenchyme to epithelial renal vesicles, which further develop into functional nephrons. FGFRL1 knockout mice lack metanephric kidneys and do not express any fibroblast growth factor (FGF) 8 in the metanephric mesenchyme, suggesting that FGFRL1 and FGF8 play a decisive role during kidney formation. FGFRL1 consists of three extracellular immunoglobulin (Ig) domains (Ig1-Ig2-Ig3), a transmembrane domain and a short intracellular domain. We have prepared the extracellular domain (Ig123), the three individual Ig domains (Ig1, Ig2, Ig3) as well as all combinations containing two Ig domains (Ig12, Ig23, Ig13) in recombinant form in human cells. All polypeptides that contain the Ig2 domain (Ig123, Ig12, Ig23, Ig2) were found to interact with FGF8 with very high affinity, whereas all constructs that lack the Ig2 domain (Ig1, Ig3, Ig13) poorly interacted with FGF8 as shown by ELISA and surface plasmon resonance. It is therefore likely that FGFRL1 represents a physiological receptor for FGF8 in the kidney and that the ligand primarily binds to the Ig2 domain of the receptor. With Biacore experiments, we also measured the affinity of FGF8 for the different constructs. All constructs containing the Ig2 domain showed a rapid association and a slow dissociation phase, from which a K_D_ of 2–3 × 10^−9^ M was calculated. Our data support the hypothesis that binding of FGF8 to FGFRL1 could play an important role in driving the formation of nephrons in the developing kidney.

## 1. Introduction

The fibroblast growth factor (FGF) signaling system controls many vital processes of our bodies, such as development, organogenesis, angiogenesis and wound healing [1,2,3,4]. In humans and mice, the system consists of 22 different FGF ligands and 4 different fibroblast growth factor receptors (FGFRs). The ligands can be grouped into 7 subfamilies, the FGF1 subfamily (FGFs 1, 2), the FGF4 subfamily (FGFs 4, 5, 6), the FGF7 subfamily (FGFs 3, 7, 10, 22), the FGF8 subfamily (FGFs 8, 17, 18), the FGF9 subfamily (FGFs 9, 16, 20), the endocrine FGF subfamily (FGFs 15/19, 21, 23; FGF19 is the human orthologue of mouse FGF15), and the subfamily of the FGF homologous factors with unrelated function (FGFs 11, 12, 13, 14) [3]. All FGFs are monomeric proteins of 17 to 34 kDa that bind to heparan sulfate and heparan sulfate containing proteoglycans, whereby their activity is dramatically increased. Together with the heparan sulfate, they interact with the receptors and induce their dimerization. A complex of two FGFs, two FGFRs and two heparan sulfate chains appears to be the active signaling complex at the cell membrane [4]. Once activated the receptors use at least four pathways to transmit the signal, namely the MAP kinase pathway, the PI3K/Akt pathway, the Jak/Stat pathway and the PLCγ pathway [1,2,3,4].

The FGFRs consist of three extracellular Ig domains (Ig1, Ig2, Ig3), a transmembrane domain and an intracellular tyrosine kinase domain, which upon activation becomes phosphorylated at multiple sites [1,2,3,4]. In the year 2000 we discovered an additional FGFR, which is now called FGFR-like protein 1 (FGFRL1) [5,6] or FGFR5 [7]. The novel receptor contains three extracellular Ig domains and a transmembrane domain similar to the classical receptors. However, in contrast to the classical receptors, it lacks the intracellular tyrosine kinase domain, which would be required for downstream signaling by transphosphorylation, but instead contains a short histidine-rich domain. This histidine-rich domain does not appear to have an important function since it can be deleted without affecting phenotype and lifespan of mutant mice [8]. The novel receptor is expressed at relatively high levels in cartilage, muscles and the kidneys and at low levels in virtually all other tissues of the body [9]. The function of FGFRL1 has been studied with the help of transgenic animals. FGFRL1 knockout mice were found to develop quite normally to term, but died immediately after birth by suffocation because they could not inflate their lungs [10]. Such mice displayed two major alterations when compared to wildtype mice; they had a malformed, underdeveloped diaphragm [10], which may explain the perinatal death, and they lacked metanephric kidneys [11].

In mice, the metanephric kidneys develop from two tissues, the ureteric bud and the metanephric mesenchyme [12,13,14,15]. At developmental stage E10.5 the ureteric bud invades the metanephric mesenchyme and starts to branch in a stereotypical fashion. The tips of the ureteric bud induce the metanephric mesenchyme to condense around the tips and form the cap mesenchyme. Some cells of the cap mesenchyme undergo a mesenchymal-to-epithelial transition and develop into renal vesicles, which further differentiate into functional nephrons [16]. In FgfrL1-deficient mice, the ureteric bud still invades the metanephric mesenchyme and branches once or twice, but then the branching process stops [11,17]. Detailed investigation of the knockout mice suggested that nephrogenesis stops because the condensed mesenchyme fails to express Lhx1, Wnt4 and Fgf8, three well-known regulators of the mesenchymal-to-epithelial transition. Consequently, no nephrons develop and the kidney rudiment remain abnormally small [11].

FGF8 has been described as an important morphogen that is required for the formation and patterning of the vertebrate embryo [18]. In the brain it acts—together with other molecules—as the “isthmic organizer” at the midbrain-hindbrain region [19,20]. In the kidney, FGF8 is specifically required for the formation of nephrons [21,22]. When FGF8 is missing, nephrogenesis stops at the renal vesicle stage and progenitor cells of the peripheral zone in the kidneys die by apoptosis. Since FGF8 is involved in gastrulation, conditional knockout mice had to be generated to study the function of FGF8 in the metanephric kidneys. Such knockout mice have abnormally small kidneys and die shortly after birth. The mutant kidneys show reduced branching and a specific lack of Wnt4 and Lhx1 expression [21,22] as found with FgfrL1-deficient kidneys [11]. Since Fgf8 is usually expressed in pretubular aggregates and renal vesicles [21,22], very similar to FgfrL1 [23], and since the phenotypes of conditional Fgf8 knockout mice [21,22] resemble that of FgfrL1 deficient mice [11,17], it has been speculated that Fgf8 might represent the physiological ligand for FgfrL1 in the kidney [15,24].

In a previous study, we had used a simple dot blot assay to identify ligands that might interact with FGFRL1 [25]. A particularly strong interaction was observed in these experiments with FGF3 and FGF8. Here we further studied the interaction of FGF8 and FGFRL1, employing several truncated versions of the novel receptor and an enzyme-linked immunosorbent assay (ELISA) as well as surface plasmon resonance to map the binding site of FGF8 in FGFRL1.

## 2. Materials and Methods

### 2.1. DNA Constructs

Constructs coding for the different Ig domains of human FGFRL1 were prepared by PCR from a previously described cDNA clone (AJ277437 [5]) using synthetic DNA primers (Microsynth, Balgach, Switzerland) as specified in Supplementary Table S1. The codons for six histidine residues as well as a stop codon were included in the downstream primers to allow purification of the expressed polypeptides by metal affinity chromatography. The technique of overlap PCR was used to generate constructs with a deletion [26]. The sequence for the signal peptide was included in all constructs to guarantee proper secretion from human cells. The PCR products were cloned into the expression vector pcDNA3.1 (Invitrogen, Carlsbad, CA, USA) harboring the gene for puromycin resistance. Authenticity and reading frame of all constructs were verified by direct DNA sequencing. The final constructs coded for the following amino acid residues (aa) of human FGFRL1: Ig123 aa 1–359; Ig12 aa 1–239; Ig13 aa 1–144, 240–357; Ig23 aa 1–26, 118–357; Ig1 aa 1–118; Ig2 aa 1–26, 118–239; and Ig3 aa 1–29, 238–359. Wherever possible, the boundaries of the individual constructs were chosen according to exon splice boundaries of the FGFRL1 gene. Domains encoded by complete exons should independently fold into the correct 3D structures according to the theory of exon shuffling.

### 2.2. Expression of Recombinant Proteins

The cDNA constructs were transfected into HEK293 cells (CRL-1573, ATCC, Manassas, VA, USA) using lipofectamine according to the supplier’s instructions (Invitrogen). Transfected cells were propagated in Dulbecco’s modified Eagle’s medium (DMEM) supplemented with 10% fetal bovine serum, nonessential amino acids, penicillin (100 u/mL) and streptomycin (100 μg/mL) (all from Sigma-Aldrich, St. Louis, MO, USA). Selection with puromycin (2.5 μg/mL, Invivogen, San Diego, CA, USA) was started after 24 h and continued until resistant colonies became visible (4–6 weeks). Several resistant colonies were pooled into a single oligoclonal batch. Subcultures from this batch were grown to confluence, rinsed with phosphate buffered saline (PBS) and cultivated in serum-free medium for 3 days. The conditioned media of the cultures were collected, supplemented with 1 mM phenylmethylsulfonyl fluoride (PMSF, Sigma-Aldrich) and dialyzed at 4 °C against PBS pH 8.0 (4 changes during 2 days). The solution was brought to 15 mM imidazole and His-Select^TM^ Nickel affinity beads (Sigma-Aldrich) were added. The suspension was incubated at room temperature for 60 min before the beads were washed with the same buffer. Specifically bound proteins were eluted from the beads with 0.5 M imidazole in PBS pH 7.4 and dialyzed against HBS-EP+ (see below) in Slide-A-Lyzer mini dialysis units (Life Technologies, Thermo Fisher Scientific, MWCO 7000, Basel, Switzerland). The size and purity of the recombinant proteins were analyzed on 12.5% SDS polyacrylamide gels stained with Coomassie blue. The molecular mass of the recombinant polypeptides without signal peptide and without/with complete glycosylation, assuming a mass of 2500 per carbohydrate chain attached to Ser/Thr-X-Asn [27], are as follows: Ig123 38.5/48.5 kDa, Ig12 24.8/29.8 kDa, Ig23 27.7/35.2 kDa, Ig13 27.0/34.5 kDa, Ig1 11.9/14.4 kDa, Ig2 15.2/17.7 kDa, and Ig3 15.4/20.4 kDa.

### 2.3. ELISA

Human FGF8b (16277-HNAE, MW 22.5 kDa) was obtained from Sino Biological (Wayne, PA, USA) and reconstituted in PBS. Compared to FGF8a, this isoform has the same amino acid sequence but includes another 11 amino acids at the N-terminus. Polystyrene 96 well microtiter plates (Sarstedt, Nümbrecht, Germany) were coated overnight with the FGF8 solution at 1 μg/mL. Residual sites of the plates were blocked with 0.05% Tween 20 in PBS. Recombinant FGFRL1 polypeptides were incubated on the coated plates at serial two-fold dilutions starting from 1 μM. After incubation for 1 h at 37 °C, the plates were washed with 0.05% Tween 20 in PBS and incubated with a humanized monoclonal antibody against human FGFRL1 [28]. This antibody has been shown to recognize an epitope residing in the Ig3 domain of FGFRL1 [29]. The plates were washed as above and incubated with phosphatase-conjugated secondary antibodies against human IgG (Sigma-Aldrich, 1:4000). After an additional washing cycle, 1 mg/mL of the phosphatase substrate p-nitrophenyl phosphate (pNPP, S0942, Sigma-Aldrich) dissolved in 1 M ethanolamine, 0.5 mM MgCl_2_, pH 9.8 was added and the enzyme reaction was allowed to proceed for 90–120 min at room temperature. Finally, the absorption of the yellow reaction product was determined at 405 nm using an Infinite M200 microplate reader (Tecan AG, Männedorf, Switzerland).

### 2.4. Surface Plasmon Resonance Analysis

FGF8 (see above) was immobilized on the surface of a biosensor chip according to the instructions of the supplier (Biacore, Uppsala, Sweden). For this purpose, a carboxymethylated matrix-free C1 chip (BR100540, GE Healthcare Life Sciences, Glattbrugg, Switzerland) was used because FGFRL1 has been demonstrated to interact with the carboxymethylated dextran matrix, which is included on regular CM5 chips [25]. The chip was activated with a 1:1 mixture of N-hydroxysuccinimide (NHS, 0.4 M) and N-ethyl-N’-dimethylaminopropyl carbodiimide (EDC, 0.1 M) at a flow rate of 5 μL/min. FGF8 (25 μg/mL) was covalently attached to the chip at pH 7.4 in HBS-EP+ (10 mM HEPES, 150 mM NaCl, 3 mM EDTA, 0.05% *v*/*v* Surfactant P20) to a total of 295 response units. This immobilization level would give a theoretical Rmax of 186–625 based on the molecular masses of the analytes. Remaining sites of the chip were blocked with 40 μL of 1 M ethanolamine pH 8.5. The reference flow cell was treated in the same way, but FGF8 was omitted in the HBS-EP+ buffer. The chip was analyzed in a X100 instrument (Biacore, Uppsala, Sweden). Increasing concentrations of FGFRL1 polypeptides diluted in HBS-EP+ were injected over the chip surface at a flow rate of 30 μL/min. After 120 s, HBS-EP+ buffer alone was passed over the chip for 240 s to monitor the dissociation phase. At the end of each measurement, the chip was regenerated for 90 s with 2 M NaCl, 100 mM sodium acetate, pH 4.5. Measurements with construct Ig123 were performed 6× in total, those with construct Ig23 3×, and those with constructs Ig12, Ig13, Ig1, Ig2 and Ig3 2× each.

All data were processed with the Biacore X100 evaluation software (Biacore, Uppsala, Sweden). The response of the reference flow cell was subtracted from the response of the FGF8 containing flow cell. The resulting sensorgrams were used to determine kinetic parameters by globally fitting the curves of the association and dissociation phases to a 1:1 binding model. Five to six different concentrations of the analyte were used to determine the parameters for each interaction. To check the quality of each fit, Chi^2^ was employed. Fitted curves were judged to be accurate when Chi^2^ was less than 5% of Rmax.

## 3. Results

### 3.1. Protein Expression

**Figure 1:**
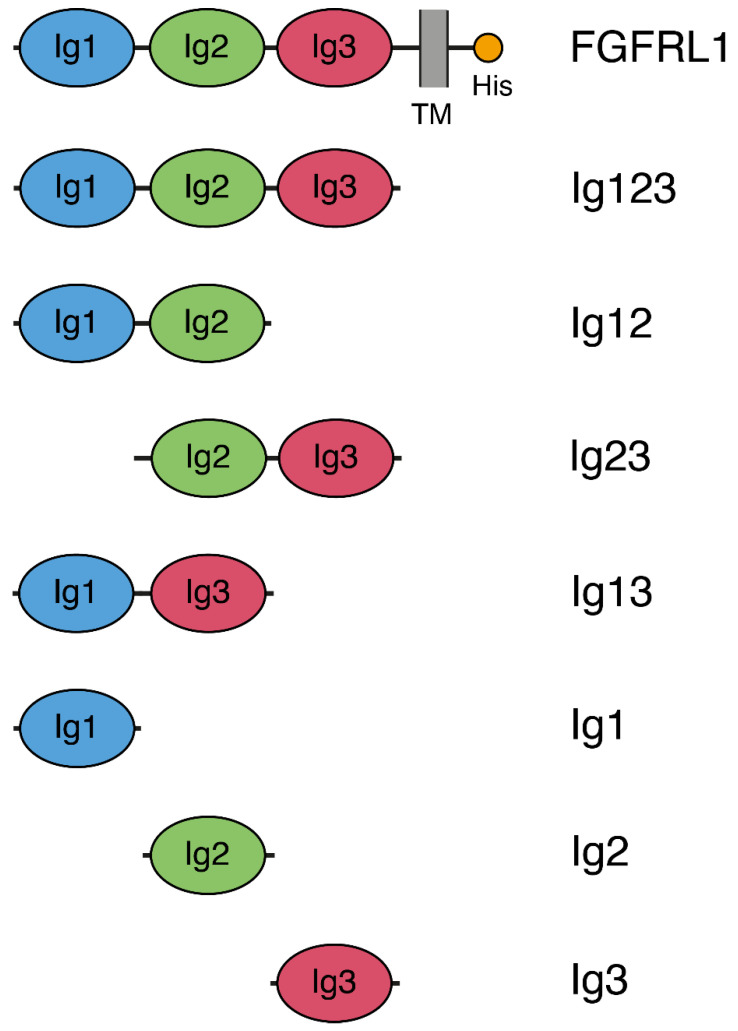
Schematic drawing of protein constructs expressed in human cells. The full-length fibroblast growth factor receptor-like protein 1 (FGFRL1) is indicated at the top with three Ig domains, a transmembrane helix (TM) and a short, histidine-rich intracellular domain (His). The entire extracellular domain (Ig123), all combinations containing two Ig domains (Ig12, Ig23, Ig13) as well as all individual Ig domains (Ig1, Ig2, Ig3) were expressed in HEK293 cells.

Seven different constructs were prepared to study the interaction of the individual domains from FGFRL1 with FGF8 (Figure 1): The complete extracellular domain of FGFRL1 (Ig123), all combinations containing two Ig domains (Ig12, Ig23, Ig13) and all three individual Ig domains (Ig1, Ig2, Ig3). All constructs could readily be expressed in HEK293 cells as recombinant proteins, but the Ig2 construct consistently gave poor yields.

**Figure 2:**
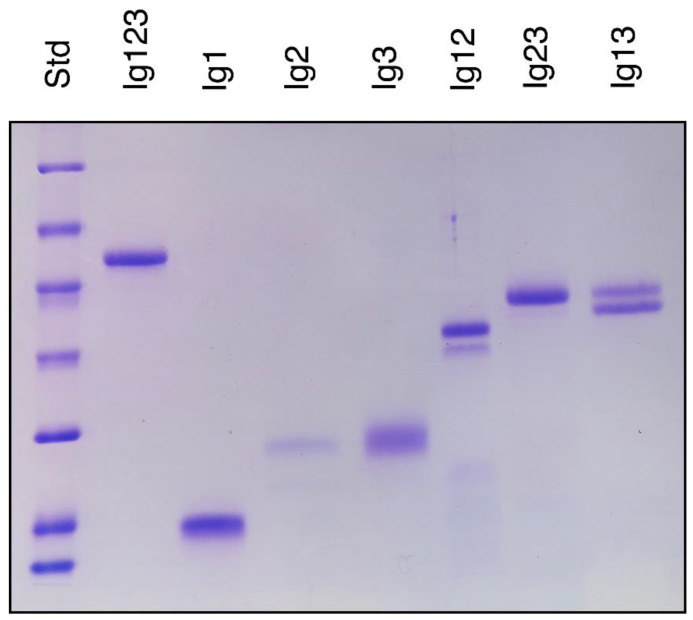
Polyacrylamide gel displaying the individual constructs expressed in human cells. Polypeptides (3 μg/lane except for Ig2) were resolved on a 12.5% SDS polyacrylamide gel and stained with Coomassie blue. In the case of Ig2, only 0.8 μg/lane was loaded due to the consistently low yields obtained with this construct. Standard proteins are included at the left-hand side with molecular masses of 116, 66, 45, 35, 25, 18.4 and 14.4 kDa. The figure shows the results from one representative experiment out of three. Note that only Ig12 and Ig13 migrate as a closely spaced doublet, suggesting incomplete glycosylation, whereas the other polypeptides migrate as single bands.

On a polyacrylamide gel, the expressed proteins migrated roughly with the expected molecular mass (Figure 2) considering that FGFRL1 contains four consensus N-glycosylation sequences Ser/Thr-X-Asn, all of which have been shown to be modified by complex carbohydrate chains [25,27]. One of these motifs is found in Ig1, one in Ig2 and two in Ig3 [5]. Consequently, all recombinant polypeptides could theoretically yield multiple bands on an SDS PAGE, depending on how many of these sites are modified. However, most of our constructs migrated as one major band. Only the Ig12 and the Ig13 constructs produced a closely spaced doublet suggesting incomplete glycosylation (Figure 2).

### 3.2. Interaction by ELISA

**Figure 3:**
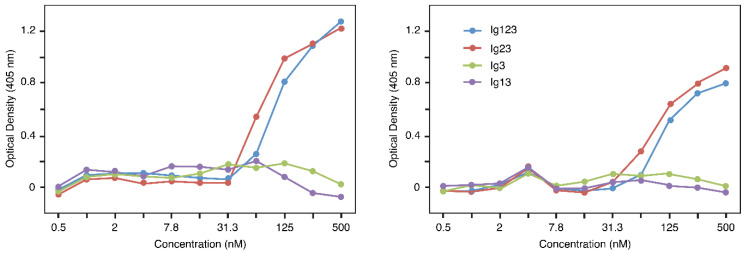
ELISA demonstrating binding of FGFRL1 constructs to fibroblast growth factor (FGF) 8. Microtiter plates were coated with FGF8 and twofold serial dilutions of four different FGFRL1 polypeptides were incubated in the wells. Bound polypeptides were detected with a monoclonal antibody against human FGFRL1, followed by a secondary enzyme-conjugated antibody. The figure shows two representative examples from a total of three assays that yielded very similar results. The color reaction was allowed to proceed for 120 min in the assay shown at the left, for 90 min in the assay shown at the right. Note that Ig123 and Ig23 bind with similar affinity to FGF8.

As a first step, a simple ELISA was used to determine a potential interaction of the constructs with FGF8. For this purpose, the wells of a microtiter plate were coated with FGF8 and different FGFRL1 constructs were incubated in the wells. Bound constructs were detected with a monoclonal antibody against FGFRL1, followed by a phosphatase-conjugated secondary antibody. After development with substrate, a strong interaction was observed between FGF8 and Ig123 as well as between FGF8 and Ig23, but no interaction could be detected between FGF8 and Ig13 or Ig3 (Figure 3). Constructs lacking Ig3, such as Ig12, Ig1 and Ig2, could not be tested with this assay because our monoclonal antibody specifically reacts with an epitope in the Ig3 domain of FGFRL1 [29]. Similar titration curves were obtained with the Ig123 and the Ig23 construct, suggesting that the two polypeptides bound with similar affinity to FGF8. Our ELISA data demonstrate that FGF8 interacts primarily with the Ig2 domain of FGFRL1, but not with the Ig1 or the Ig3 domain.

### 3.3. Biacore Experiments

**Figure 4:**
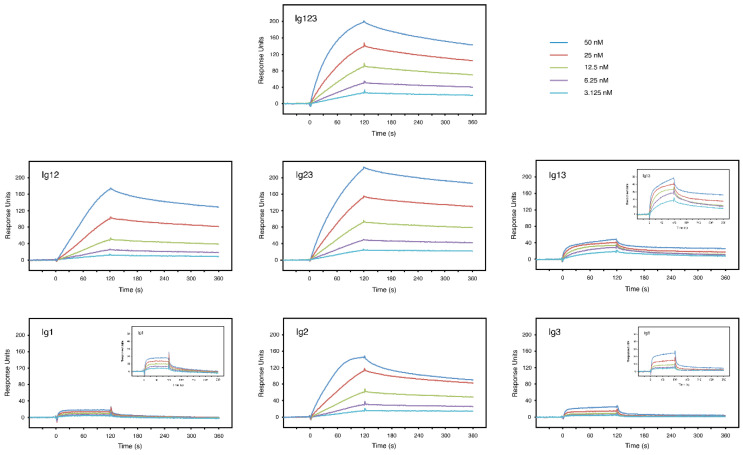
Surface plasmon resonance analysis of FGF8 and different FGFRL1 constructs. A Biacore sensor chip was coated with FGF8 and increasing concentrations of different FGFRL1 polypeptides as indicated were injected over the chip surface. After 120 s, buffer alone was passed over the chip to monitor the dissociation phase. Depending on the individual construct, the assay was performed 2–6 times yielding very similar results and the panels show one representative example of each measurement. In order to visualize low affinity interactions, small insets are displayed, in which the range of the *y*-axis was enlarged (0–50 RU). Note that Ig123, Ig12, Ig23 and Ig2 bind with high affinity to FGF8 immobilized on the chip, whereas all of the other constructs show dramatically lower binding responses. The different concentrations were: Blue 50 nM, red 25 nM, green 12.5 nM, purple 6.25 nM, light blue 3.125 nM.

Surface plasmon resonance was utilized for more quantitative measurements. FGF8 was covalently attached to the surface of a Biacore sensor chip and increasing concentrations of the individual constructs were injected over the chip surface. Typical interaction curves showing a fast association rate and a slow dissociation rate were obtained with the Ig123 construct, which contains all three Ig domains (Figure 4). Similar binding curves were also observed with the Ig12 and the Ig23 constructs containing only two Ig domains. Moreover, a robust interaction was even noted with the Ig2 construct alone. In sharp contrast, the Ig1 and the Ig3 constructs barely interacted with FGF8 and the Ig13 construct showed only very poor binding (Figure 4). To better visualize these weak interactions, the *y*-axis of the sensorgrams was enlarged (Figure 4, small insets). In this way, it became evident that Ig1 and Ig3 bound rapidly to the chip surface and reached a plateau within seconds, which is unusual for a physiological interaction, but rather characteristic of a high bulk contribution. Moreover, the Ig13 construct showed a very complex interaction with FGF8, which was clearly distinct from those of Ig1 and Ig3 alone.

The data shown in Figure 4 were used to calculate specific dissociation constants for the analyte/ligand interactions (Table 1). A kinetic approach was used and the curves were fitted to a 1:1 binding model. The raw data and fitted curves are shown in Supplementary Figure S1. A K_D_ of 3.4 × 10^−9^ M was calculated for the FGF8/Ig123 complex, K_D_s of 1.9 × 10^−9^ M and 2.1 × 10^−9^ M for the FGF8/Ig12 and FGF8/Ig23 complexes, respectively, and a K_D_ of 1.7 × 10^−9^ M for the FGF8/Ig2 complex. Chi^2^, which was used as a measure for the goodness of fitting, was found to be only 0.5–1.2% of Rmax, indicating extremely good fits. Thus, Ig123, Ig12, Ig23 and Ig2 show very high affinities for FGF8 (2–3 nM). When the same procedure was used for the Ig1 and Ig3 constructs, Chi^2^ was found to be 7–8% of Rmax, which indicated bad fits to a 1:1 binding model (Table 1). With the Ig13 construct, Chi^2^ was even found to be 27% of Rmax. It is therefore likely that the constructs Ig1, Ig3 and Ig13 undergo complex interactions with FGF8 that do not conform to a 1:1 binding model. Kinetic data drawn from the model are therefore unreliable in these cases. Since construct Ig123 with three Ig domains conforms well to the 1:1 binding model and gives reliable data, but constructs Ig1, Ig3 and Ig13 do not, it is likely that the latter interactions reflect unspecific, nonphysiological bindings. More studies would be required to exactly elucidate the nature of these complex, potentially artificial interactions. Taken together, our data clearly indicate that FGF8 binds primarily to the Ig2 domain of FGFRL1 and that the Ig1 and Ig3 domains have only minor effects on affinity.

## 4. Discussion

The major function of FGFRL1 during mammalian development is the promotion of nephrogenesis as demonstrated by several published studies [11,17,24]. This function appears to be accomplished by inducing mesenchymal cells from the cap mesenchyme to undergo a mesenchymal-to-epithelial transition to renal vesicles and by inhibiting cell death in the nephrogenic zone.

In this study, we observed a strong interaction between FGF8 and FGFRL1. This interaction had previously been demonstrated by a dot blot assay where FGFRL1 bound to FGF8 immobilized on a nitrocellulose membrane [25]. It was confirmed and quantitated in this publication by ELISA where FGFRL1 bound to FGF8 immobilized on a microtiter plate (Figure 3), and by surface plasmon resonance where FGFRL1 interacted with FGF8, which had been covalently attached to the surface of a Biosensor chip (Figure 4). During kidney development, FGFRL1 and FGF8 show a similar spatio-temporal expression pattern in pretubular aggregates, renal vesicles and nascent nephrons [11,21,22,23]. Moreover, *FgfrL1* knockout mice and conditional *Fgf8* null mice display very similar phenotypes with abnormally small kidneys, reduced ureteric branching and increased cellular apoptosis in the cortical zone [11,17,21,22]. It is therefore likely that FGFRL1 represents a physiological receptor for FGF8 in the kidney.

The biosensor experiments allowed us to measure the binding affinity between FGF8 and FGFRL1. Affinities between 2 × 10^−9^ M and 3.3 × 10^−9^ M were observed for different FGFRL1 constructs (Figure 4). These values are comparable to the affinity previously reported for the FGFRL1/FGF3 complex (4.2 × 10^−9^ M) [25], but they are about 100-fold higher than the affinities reported for the interactions between any FGF ligand and the classical receptors FGFR1-FGFR4 [30,31]. For example, a K_D_ of 1.3 × 10^−7^ M has been reported for the FGF8/FGFR2 complex [31]. K_D_ values similar to those reported herein have been found only with mutant receptors that are associated with craniosynostosis syndromes [30]. For example, a K_D_ of 2.4 × 10^−8^ M has been reported for the FGF2/FGFR1 complex from a patient with Pfeiffer syndrome [30]. It should be emphasized, however, that our study cannot directly be compared to previous studies with the classical FGFRs because we used constructs that had been expressed by human cells in culture. Our polypeptides should therefore include the physiological modification with carbohydrates, in contrast to the constructs used in the literature [30,31,32], which had been expressed by bacteria and thus are not glycosylated.

In the case of the classical receptors FGFR1 and FGFR2, the FGF ligands are known to bind into a pocket, which is formed by the Ig2 and the Ig3 domain [4]. The exact binding sites have been characterized for the FGF1/FGFR2 and the FGF2/FGFR1 complex by X-ray crystallography [32]. Three major sites have been identified: (i) a generic binding site in the Ig2 domain, (ii) a site in the linker region between Ig2 and Ig3 and (iii) a site in Ig3, which appears to determine the specificity of the receptors with a particular ligand. In our case with FGFRL1, all constructs that contain the Ig2 domain (Ig123, Ig23, Ig12, Ig2) interacted with FGF8 with comparable affinities (K_D_ 2–3 × 10^−9^ M). The major binding site must therefore reside within the Ig2 domain. It is likely that the Ig3 domain contributes only little to this affinity because all constructs containing the Ig3 domain, but lacking the Ig2 domain, showed poor binding (Figure 4). Moreover, the linker between Ig2 and Ig3 (aa 240–260) cannot add much to the binding strength because the constructs Ig12 and Ig2, which are lacking this linker, showed an affinity similar to the constructs Ig23 and Ig123, which include the linker.

By a direct comparison of the amino acid sequences from the classical receptors and that from FGFRL1 we may predict the sequence of the principal binding site [6]. In FGFR1, this site was elucidated by 3D analysis as 165-LHAVPA-170, where leucine-165, alanine-167 and proline-169 are involved in the formation of hydrophobic contacts with the ligand [32]. This sequence is fully conserved in FGFR2 as 166-LHAVPA-171. In FGFRL1, the corresponding sequence is 159-VIARPA-164, where alanine-161 and proline-163 are conserved. It is therefore possible that this sequence from FGFRL1 is involved in FGF8 binding. To confirm our hypothesis, experiments with mutated FGFRL1 proteins would be required, in which alanine-161 and proline-163 would be substituted by other residues.

What is the effect elicited by FGF8 binding to FGFRL1 in the kidney? FGFRL1 cannot signal by itself as it does not contain any tyrosine kinase activity in the intracellular domain [5]. Moreover, knockout experiments with mice demonstrated that the intracellular domain of FGFRL1 does not serve a major function since deletion did not affect the phenotype and lifespan of the mutant mice [8]. One possibility would be that the FGF8/FGFRL1 complex will interact with another receptor, which in turn is capable of downstream signaling. In the developing kidney, only FGFR1 shows a similar spatio-temporal distribution, whereas FGFR2-FGFR4 display quite different expression patterns [33]. We have tried to demonstrate an interaction of the FGF/FGFRL1 complex with FGFR1, but, so far, we have not found such an interaction. Another possibility would be that FGFR1 and FGFRL1 compete for the same ligand in the developing kidney. In most tissues of the body, the expression levels of FGFR1 are much higher than those of FGFRL1 [6]. When the relative concentration of FGFR1 is high, FGF8 might primarily bind to FGFR1 and induce cell proliferation by transphosphorylation of the intracellular domain and downstream signaling. However, when the FGFR1 concentration is low, FGF8 might primarily bind to FGFRL1 due to its stronger affinity for this receptor. The activated receptor complex could then induce the mesenchymal-to-epithelial transition described above by an unknown mechanism. It is conceivable that a direct interaction of FGFRL1 expressing cells is required for this effect as recently demonstrated with CHO cells [34]. When overexpressed in such cells, FGFRL1 was able to induce the formation of large syncytia containing dozens of nuclei and this activity was found to depend on the direct contact with FGFRL1 expressing cells [34].

## 5. Conclusions

Taken together with evidence from the literature, our results suggest that FGFRL1 is a physiological receptor for FGF8 in the kidney and that FGF8 binds primarily to the Ig2 domain of FGFRL1. The interaction of FGF8 with FGFRL1 is in the nanomolar range (2–3 nM). Our data are consistent with the hypothesis that the FGF8/FGFRL1 complex regulates development of nephrons by controlling the mesenchymal-to-epithelial transition in the metanephric mesenchyme. Further work will be required to fully elucidate the underlying molecular mechanisms.

## Figures and Tables

**Table 1:** Summary of kinetic data.

Construct	ka (1/Ms)	kd (1/s)	K_D_ (M)	Rmax (RU)	tc	Chi^2^ (RU^2^)	*U*−Value
Ig123	3.73 × 10^5^	1.26 × 10^−3^	3.37 × 10^−9^	224.20 (0.33)	6.257 × 10^17^	3.00	1
	(9.0 × 10^2^) *	(6.8 × 10^−6^)					
Ig12	5.49 × 10^6^	1.07 × 10^−2^	1.94 × 10^−9^	192.10 (0.84)	1.107 × 10^7^	4.02	12
	(6.1 × 10^5^)	(1.0 × 10^−3^)					
Ig23	3.90 × 10^5^	8.06 × 10^−4^	2.07 × 10^−9^	264.00 (0.60)	7.533 × 10^7^	1.43	1
	(4.5 × 10^3^)	(5.7 × 10^−6^)					
Ig13	1.59 × 10^6^	2.04 × 10^−3^	n.a. **	37.47 (0.31)	8.027 × 10^17^	10.10	5
	(2.2 × 10^4^)	(5.9 × 10^−5^)					
Ig1	2.25 × 10^6^	2.98 × 10^−2^	n.a.	17.02 (0.41)	1.863 × 10^14^	1.18	7
	(1.1 × 10^5^)	(7.2 × 10^−4^)					
Ig2	5.32 × 10^6^	9.20 × 10^−3^	1.73 × 10^−9^	143.50 (0.18)	1.421 × 10^7^	1.13	3
	(1.5 × 10^5^)	(2.5 × 10^−4^)					
Ig3	3.09 × 10^5^	5.27 × 10^−3^	n.a.	14.75 (0.49)	4.606 × 10^7^	1.24	7
	(4.5 × 10^4^)	(6.2 × 10^−4^)					

* numbers in brackets give standard errors. ** n.a. = not applicable since Chi^2^ > 5% of Rmax.

## Supplementary Materials

The following are available online at https://www.mdpi.com/2218-273X/10/10/1399/s1, Table S1: Primers used to prepare cDNA clones, Figure S1: Kinetic fit of the binding data.
