# Supplementary material for: Dissecting the Interaction of FGF8 with Receptor FGFRL1
Source: Biomolecules. 2020 Oct 1;10(10):1399. doi: 10.3390/biom10101399 (PMC7600612; doi:10.3390/biom10101399)
Supplement: Supplementary file 1 [file biomolecules-10-01399-s001.pdf]

biomolecules

Supplementary Materials

Dissecting the Interaction of FGF8 with Receptor FGFRL1

Lei Zhuang [1], Monique Vogel [1], Peter M. Villiger [2] and Beat Trueb [1,*]

[1] Department for BioMedical Research, University of Bern, 3008 Bern, Switzerland; lei.zhuang@dbmr.unibe.ch (L.Z.); monique.vogel@dbmr.unibe.ch (M.V.)
[2] Department of Rheumatology, University Hospital, 3010 Bern, Switzerland; peter.villiger@insel.ch
* Correspondence: beat.trueb@dbmr.unibe.ch

Table S1. Primers used to prepare cDNA clones (5'-3')*.

Ig123	
Up	CCGGATCCGGACAGGCCGAGATG
Low	CACGAATTCAGTGGTGATGGTGATGATGTGGGTCTGGCAGCACGGT
Ig1	
Up:	CCGGATCCGGACAGGCCGAGATG
Low:	CACGAATTCAGTGGTGATGGTGATGATGATCCAGCACGACGAGGGT
Ig2	
Up:	CCGGATCCGGACAGGCCGAGATG
Low:	CACGAATTCAGTGGTGATGGTGATGATGGATCACATCCACCTTGTA
Ig2 overlap signal-Ig2	
Up:	GCCGCCGCCCGAG'ATGACATTAGCCCAGGGAAG
Low:	TGGGCTAATGTCAT'CTCGGGCGGCGGCGGC
Ig3	
Up:	CCGGATCCGGACAGGCCGAGATG
Low:	CACGAATTCAGTGGTGATGGTGATGATGTGGGTCTGGCAGCACGGT
Ig3 overlap signal-Ig3	
Up:	CGAGGCCCCCC'TGTGATCCAGCGGACCCG
Low:	CCGCTGGATCACA'GGGGGGCCTCGGGCGGC
Ig12	
Up:	CACGAATTCCCGGACAGGCCGAGATG
Low:	GAAGGATCCTCAGTGATGGTGATGGTGATGGGGTGGGATCACATCCACCTTGTA
Ig13	
Up:	CACGAATTCCCGGACAGGCCGAGATG
Low:	GAAGGATCCTCAGTGATGGTGATGGTGATGGGGTGGCAGCACGGTGAGGA
Ig13 overlap Ig1-Ig3	
Up:	AGCCAGCAGTGG'CAGCGGACCCGTTCCAAG
Low:	ACGGGTCCGCTG'CCACTGCTGGCTGGCGGG
Ig23	
Up:	CACGAATTCCCGGACAGGCCGAGATG
Low:	GAAGGATCCTCAGTGATGGTGATGGTGATGGGGTGGCAGCACGGTGAGGA
Ig23 overlap signal-Ig2	
Up:	GCCGCCGCCCGAG'ATGACATTAGCCCAGGGAAG
Low:	TGGGCTAATGTCAT'CTCGGGCGGCGGCGGC

* Underlined residues mark the restriction sites used for subcloning.

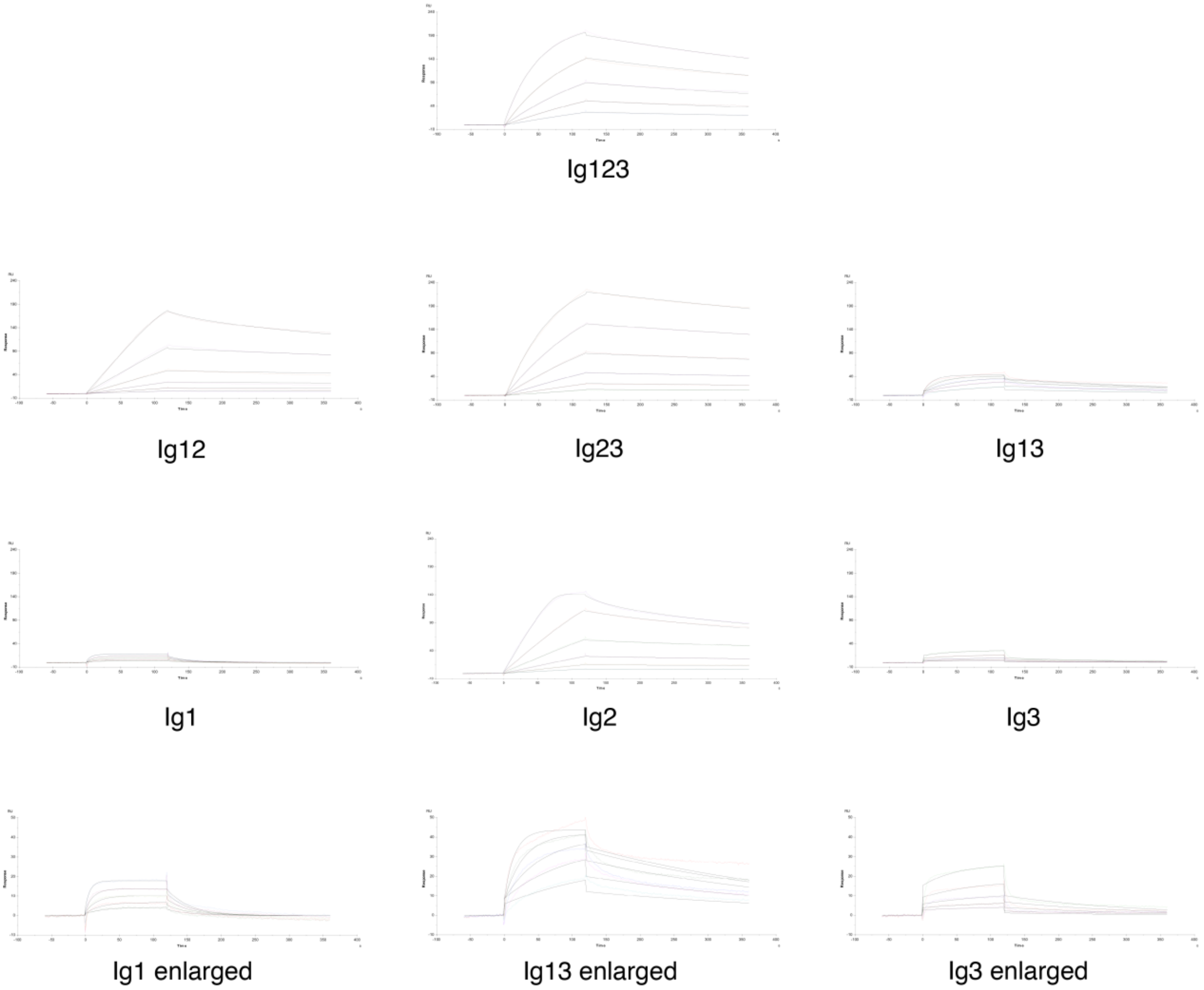

Figure S1: Kinetic fit of the binding data.
